# B Cells in Neuroinflammation: New Perspectives and Mechanistic Insights

**DOI:** 10.3390/cells10071605

**Published:** 2021-06-26

**Authors:** Julie J. Ahn, Mohammad Abu-Rub, Robert H. Miller

**Affiliations:** 1 School of Medicine and Health Sciences, The George Washington University, Washington, DC 20037, USA; jahn724@gwu.edu; 2 Medical Faculty Associates, The George Washington University, Washington, DC 20037, USA; aburub@gwu.edu

**Keywords:** B cell, neuroinflammation, neurological disorders, cytokines, multiple sclerosis, Parkinson’s disease, Alzheimer’s disease

## Abstract

In recent years, the role of B cells in neurological disorders has substantially expanded our perspectives on mechanisms of neuroinflammation. The success of B cell-depleting therapies in patients with CNS diseases such as neuromyelitis optica and multiple sclerosis has highlighted the importance of neuroimmune crosstalk in inflammatory processes. While B cells are essential for the adaptive immune system and antibody production, they are also major contributors of pro- and anti-inflammatory cytokine responses in a number of inflammatory diseases. B cells can contribute to neurological diseases through peripheral immune mechanisms, including production of cytokines and antibodies, or through CNS mechanisms following compartmentalization. Emerging evidence suggests that aberrant pro- or anti-inflammatory B cell populations contribute to neurological processes, including glial activation, which has been implicated in the pathogenesis of several neurodegenerative diseases. In this review, we summarize recent findings on B cell involvement in neuroinflammatory diseases and discuss evidence to support pathogenic immunomodulatory functions of B cells in neurological disorders, highlighting the importance of B cell-directed therapies.

## 1. Introduction

The central nervous system (CNS) was traditionally considered to be a site of strict immune privilege, since there was a perceived lack of lymphatic vessels and professional antigen-presenting cells in the brain parenchyma as well as physical barriers preventing circulating immune cells from entering the CNS. Subsequent studies redefined the concept of immune privilege within the CNS as knowledge of the peripheral immune system and CNS interactions expanded. Although the CNS continues to be considered immune privileged, it is now clear that low numbers of lymphocytes enter CNS lymphatic vessels under healthy steady-state conditions and support CNS immune surveillance [1]. One conduit of cellular dispersal through the CNS is via the cerebrospinal fluid (CSF). The choroid plexus is the main source of CSF and consists of epithelial cells that form a tight barrier separating the blood and CNS [2,3]. CSF-interstitial fluid (ISF) drainage follows specific pathways in the CNS, involving the ventricles, subarachnoid space, parenchyma, and subcortical regions, and drain into deep cervical lymph nodes [4,5]. This functional connection between the CNS and cervical lymph nodes has further supported the concept of neuroimmune crosstalk [6,7,8,9]. In an injured or inflamed CNS, lymphocytes, including T and B cells, increase by severalfold and can be found throughout the parenchyma, CSF-ISF, and perivascular and meningeal spaces due to antigens draining to lymph nodes or disruptions to the blood-brain barrier [3,8,10]. The emerging role of the adaptive immune system in the pathophysiology of neurological disorders has led to the successful development of treatments that specifically target the adaptive immune system, including B cell-depleting monoclonal antibodies. 

Of the lymphocytes present in the CSF of a healthy individual, the vast majority are CD3+ T cells that are involved in immune surveillance, and B cells account for less than 1% [10,11], leading researchers to focus the majority of their efforts on the role of T cells and T-cell subtypes in CNS immune regulation and autoimmunity. This focus has shifted in the past few years, and the concept of B cells as regulators of CNS inflammation has emerged, resulting in the development of B cell-targeted therapies for various CNS and peripheral nervous system (PNS) diseases. The mechanisms of B cell functions in pathology can be complex. They play a number of critical roles in the immune system, including antigen presentation, cytokine production, and antibody secretion, so their contributions to immune responses in the CNS can be highly diverse. 

In this review, we summarize B cell and CNS immune properties and discuss evidence supporting the emerging concept that B cells play a critical role in regulating adaptive and innate immune responses in various neurological disorders. B cells have been known to play a role in certain neuroinflammatory contexts, including neuromyelitis optica and autoimmune encephalitis where antibodies produced by B cells target CNS antigens, contributing to injury [12,13,14]. Emerging evidence suggests that a range of neurodegenerative diseases also result in immune cell activation, and in this review, we focus on B cell driven neuroinflammatory responses in diseases that have recently provided insights into our understanding of how the immune system contributes to the pathogenesis of neurological disorders, including multiple sclerosis (MS), Parkinson’s disease (PD), and Alzheimer’s disease (AD). 

## 2. B Cell Biology

Together, B cells and T cells form the adaptive immune system, and their main role is to protect the body from harmful pathogens. Foreign substances that enter the body are recognized as antigens and elicit an immune response in which B and T cells in combination with other cell types of the immune system selectively recognize and eliminate the antigens. While the B cell lineage is best known for its role in antibody generation and secretion, B cells contribute to many aspects of the adaptive immune response, including both antibody-dependent and independent functions [15,16,17]. 

B cells develop in the fetal liver and postnatal bone marrow, where they undergo several well-defined maturation and differentiation stages in response to inductive signals [17]. B cells mature from hematopoietic stem cells, which generate progenitor B cells, precursor B cells, immature naïve, transitional, and then mature naïve B cells. In the presence of a specific antigen and with the help of T follicular helper cells, the mature naïve B cells that now reside in secondary lymphoid organs are activated and differentiate into antibody-producing plasma cells and memory B cells [18]. In general, surface expression of B220 and CD19 can identify cells committed to the B cell lineage and are most often used for the histological confirmation of B cell presence in both murine and human tissues. Another common B cell surface marker, CD20, is also expressed on mature B cells but is significantly downregulated on plasma cells [19,20]. These cell surface proteins have been common targets in B cell-directed therapies, like rituximab and ocrelizumab, and ofatumumab, which target CD20-expressing B cells while leaving most antibody-producing plasma cells intact [21,22]. In addition to antibody production, mature B cells act as uniquely specialized professional antigen-presenting cells (APC). B cells have highly specific antigen processing machinery because of B cell receptor (BCR) signaling and HLA-DO expression. These characteristics allow BCR antigen presentation to be significantly more efficient and specific (up to 10,000-fold) than other APCs [23]. This highly efficient antigen-presenting capability has been harnessed for the development of B cell immunotherapies [24,25,26].

**Figure 1:**
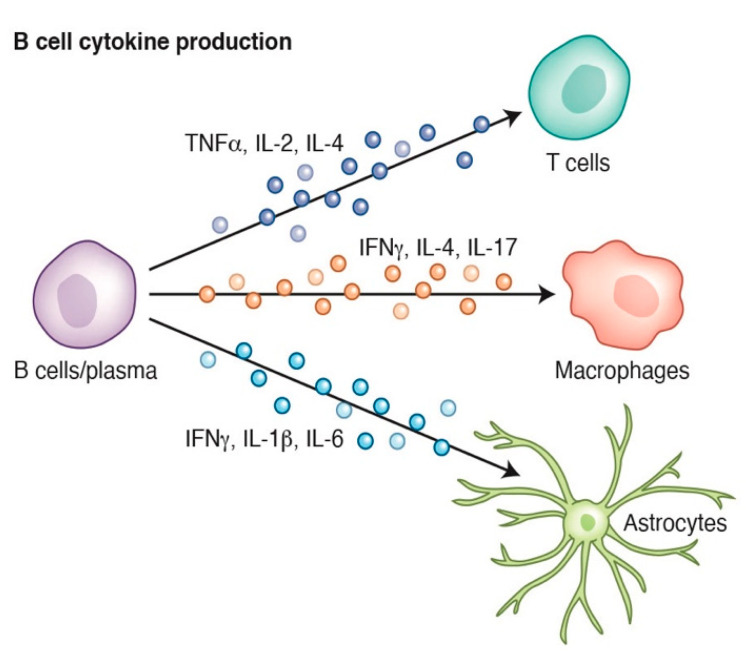
B cells produce cytokines and regulate T cell function as well as macrophage and astrocyte activation. Credit: Katie Vicari.

A major B cell effector mechanism is the selective release of a spectrum of cytokines. Naïve B cells do not secrete extensive cytokines after activation and require additional inducers from the immune microenvironment to enhance their cytokine production. The outcomes of B cell signaling such as the induction of Th1/Th2 cells depends in part on environmental cues. For example, B cell receptor signaling induces B cell proliferation and production of pro-inflammatory cytokines, such as TNFα and IL-6 [27,28,29]. Although cytokine production is relatively widespread, their selective secretion from B cells is vital for multiple aspects of immune health. For example, naïve B cells produce cytokines that bind to CD4+ T cells and influence their subsequent functionality. Bacterial and viral infections can stimulate B cell production of tumor necrosis factor (TNF) and interferon gamma (IFNγ) that subsequently promote the differentiation of T cells towards a Th1 path, inducing macrophage activation and proliferation that results in the production of more cytokines that increase the immune response under certain conditions [30]. Similarly, mice lacking B cells can develop a dysfunctional Th1 response during infection [31]. Not only are B cell-derived cytokines important for the generation of specific immune responses, they are also essential for the generation of secondary and tertiary lymphoid organs [32] and an example of such a cytokine is lymphotoxin (LT). The targets of these cytokines are not limited to the immune system, and LT was recently shown to influence CNS neural stem cell fate by promoting glial cell differentiation and suppressing neurogenesis through LTβR signaling. Moreover, in the CNS, it is well known that microglia and astrocytes represent the main responders to inflammatory stimuli, including cytokines, such as IFNγ, IL-4, IL-17, and IL-6 [33,34,35,36,37], emphasizing the critical interplay of the adaptive immune system and the CNS [38] (Figure 1).

While the production of cytokines by B cells is essential for normal homeostasis and maintenance of overall immune health, dysregulation of B cell cytokine production can be highly deleterious and result in the development of autoimmune diseases in which tolerance to self-antigens is broken and the activated immune system damages targeted tissues and organs. In the CNS, an important B cell-derived cytokine is granulocyte–macrophage colony-stimulating factor (GM-CSF), a protein that has been well-documented in many autoimmune diseases including multiple sclerosis (MS), a demyelinating neurological disease [39]. Other B cell-secreted and expressed factors, including IL-6, IL-10, IL-35, and transcription factor T-bet modulate immune responses by upregulating or downregulating production of other pro- and anti-inflammatory cytokines by additional cell types. B cell expression of T-bet, for example, is required for Th-1-dependent differentiation of B cells into antibody-secreting cells [40]. Based on these findings, targeting specific effector B cell subsets that upregulate inflammatory activity can be useful in deciphering B cell-mediated mechanisms of autoimmune diseases. 

## 3. CNS Immune Regulation

The brain and spinal cord are isolated from peripheral immune responses by two principal barriers, the blood-brain barrier (BBB) and the blood-cerebrospinal fluid (CSF) barrier, which regulate the passage of immune mediators and immune cells into the CNS. Under homeostatic conditions, leukocytes such as memory T cells, penetrate the CNS and flow through the CSF contained in the meninges, a connective tissue layer that surrounds the CNS [41,42]. The CSF and lymphatic vessels allow a minimal level of leukocyte trafficking from the peripheral immune system that results in surveillance of the CNS. In pathologic conditions, however, such as cerebrovascular or neurodegenerative diseases, leukocyte trafficking into the brain parenchyma is significantly increased, and activation of innate immune cells, such as mast cells, further compromise the integrity of the blood-brain barrier [43]. These events create an unbalanced pro- and anti-inflammatory environment [3,44,45]. 

Innate immune responses in the CNS are most often characterized by reactivity of glial cells, specifically microglia and astrocytes [46]. Along with activation of other innate immune cells such as mast cells, glial cells are capable of compromising the blood-brain barrier. Microglia, the CNS resident macrophages, can shift their functional states between a pro-inflammatory phenotype—characterized by release of inflammatory factors, antigen presentation, and expression of C1q and other markers—to a more neuroprotective phenotype—characterized by phagocytosis of debris and apoptotic cells and expression of S100B and other markers. Similarly, astrocytes display functional heterogeneity under both homeostatic and disease states that may be either neurotoxic or neuroprotective [47,48,49,50]. Together, microglia and astrocytes can adopt pro- or anti-inflammatory phenotypes to either prevent or exacerbate disease, and studies have shown that reactive microglia and astrocytes contribute to disease through upregulated transcriptional activity of pro-inflammatory genes [51,52,53]. 

## 4. B Cells in Neurological Diseases

### 4.1. Multiple Sclerosis

MS is a chronic inflammatory demyelinating disease characterized by demyelinating lesions in the brain and spinal cord. Although MS is primarily recognized as a white matter disease, both MRI and neuropathological studies of the brain have shown extensive gray matter involvement, particularly of the cortical regions, even at early stages of the disease, and the degree of gray matter involvement is correlated with physical and cognitive symptoms and becomes more fulminant in progressive forms of the disease [54,55]. In addition to demyelination, microglial and astrocyte activation as well as axonal loss are frequently associated with disease activity. While the disease onset and clinical course may differ, approximately 90% of patients present with relapsing-remitting MS (RRMS) in which patients have periods of relapse (new or worsening neurological symptoms) and remission [56,57]. After a variable number of years, for most RRMS patients, symptoms eventually worsen with no periods of remission, and this is defined as secondary progressive MS (SPMS) [56,58,59]. Individuals with accumulating disability from the onset without relapses have a variant of the disease referred to as primary progressive MS (PPMS) [21,56,60]. 

Although CNS demyelination is a hallmark of MS, genetic studies showing an association with the MHC class II HLA-DRB1*15:01 allele [61,62,63,64] suggest that MS has a critical adaptive immune system component that drives disease. Important advances in our understanding of the pathology of MS have come from the development of animal models that mimic aspects of the human disease. With the realization that MS was predominantly a disease of white matter, initial studies involved the injection of white matter preparations to provoke an immune response and subsequent pathology. While successful, the approach was inconsistent and later refinements including the use of more defined antigens combined with stimulation of the immune system with pertussis toxin resulted in the establishment of experimental allergic encephalomyelitis (EAE). While there are currently a number of variations of EAE, one of the most common models involves injection of peptides of myelin oligodendrocyte glycoprotein (MOG—a major component of CNS myelin) coupled with an adjuvant into an appropriate strain of mice [65,66,67]. This results in demyelination, predominantly in the spinal cord. The demyelination is associated with immune cell infiltration and glial activation, reminiscent of lesions in MS. Historically, MS has been considered a T cell-mediated disease in large part due to histological observations showing an abundance of T cells in demyelinating lesions [1]. Furthermore, CD4 and CD8 T cells have been shown to be present in the healthy adult brain parenchyma, where they may be retained after local infection [68]. A role for T cells in the generation of EAE pathology was further demonstrated through the adoptive transfer of myelin-specific T cells that were able to induce EAE [69]. These studies guided the field to the development of many of the therapies that are currently available, in particular interferon ß [70,71]. A potential challenge with the murine EAE models is the influence of species specificity, and to begin addressing these concerns a non-human primate model was developed in the marmoset. In a series of studies, it was shown that selective depletion of B cells resulted in significant reductions in the extent of demyelination in the marmoset EAE model [72,73]. Indeed, without B cells, white and gray matter damage was significantly reduced, suggesting a necessary role of B cells in disease progression. 

The best-known function of B cells is the production of antibodies, and traditionally, it was believed that the role of B cells in MS was the production of autoantibodies directed against myelin proteins. One of the earliest implications of a role for antibodies in MS was the presence of unique immunoglobulins (IgG) in the CSF of approximately 90% of MS patients. Transcriptomic studies later confirmed the source of these IgG’s as clonal B cell populations [74]. Though target antigens of these clonal IgG fractions have been extensively studied and candidates have been proposed, their function remains uncertain. The presence of these intrathecal IgG fractions, referred to as oligoclonal bands in antibody assays, was however, found not to be specific to MS but also present in neurological conditions, such as encephalitis and neuroborreliosis [75,76]. Unlike other conditions, such as measles, Herpes simplex virus, and Cytomegalovirus, where the antibodies are directed against epitopes from the causative agents, the significance of the antigens in MS are unknown [77,78], suggesting either that a MS-specific autoantibody has yet to be identified or more likely rather, the role of B cells in neuroinflammation is not primarily due to antibody production. 

**Figure 2:**
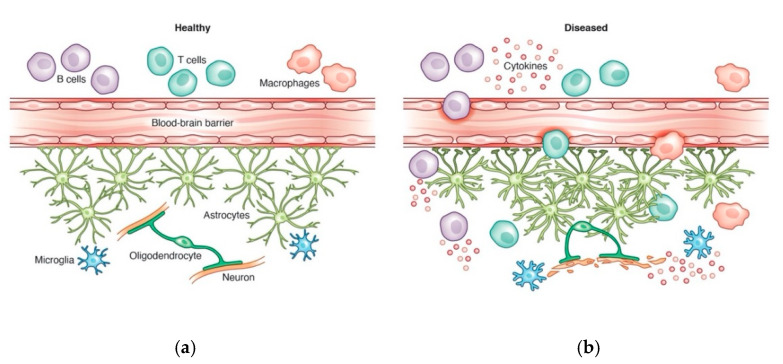
(**a**) In the healthy CNS, the blood-brain barrier prevents widespread infiltration of peripheral immune cells, including B cells, T cells, and macrophages. (**b**) In the diseased, inflamed brain, pro-inflammatory peripheral immune cells may enter the CNS through a dysfunctional blood-brain barrier, allowing cytokine release in the CNS. This increases astrocyte and microglia reactivity, which further propagates the inflammatory cascade, damaging myelin and neurons. Credit: Katie Vicari.

The hypothesis that there is an antibody-independent role for B cells in MS is supported by recent successful clinical trials using various anti-CD20 treatments, which target B cells that do not produce antibodies [21,22,79,80]. The CD20 protein is not expressed on pro-B cells or antibody-secreting plasma cells, however, a clinical trial with rituximab, a monoclonal antibody that selectively targets CD20+ B cells, reported a significant reduction in relapses and gadolinium-enhancing lesions in patients receiving rituximab compared to the placebo group [22]. Even in primary progressive MS cases, treatment with ocrelizumab, another anti-CD20 antibody, resulted in decreased disability progression and brain lesion volume [21]. Since disease progression is altered by the depletion of B cells without affecting plasma cells, the most likely interpretation is that B cells play a role in MS pathogenesis through an antibody-independent mechanism (Figure 2).

Consistent with a role for B cells in the pathology of MS is the presence of B cells within MS lesions [81] and ectopic B cell follicles in the meninges of MS patients [59,82], providing further indications that B cells may play more of a role in CNS disease than antibody production. Recent evidence suggests that B cell-derived cytokines may play a role in MS pathogenesis (Figure 2). B cells in MS patients have aberrant cytokine profiles, typically exhibiting increased ability to produce pro-inflammatory cytokines, such as IL-6 and GM-CSF, and decreased production of anti-inflammatory cytokines, such as IL-10 [28,39]. Notably, patients with MS who are treated with B cell depletion therapy do not exhibit the same skewed cytokine profiles once treatment stops and B cells return to normal levels, and neither do they experience relapses [39]. This suggests that the pro-inflammatory profile of B cells may regulate disease progression, but whether the same profile exists in both the periphery and CNS invading B cells is unclear. In a study examining CSF and peripheral blood of patients with MS, a disproportionate increase of B cells in the CSF was found in patients with active brain lesions [83]. Surprisingly, these numbers were greater than the number of T cells found in the CSF. These intrathecal B cells were found to be clonally related to peripheral blood B cells by immune repertoire analysis, suggesting that B cells invade and activate in the CNS; however, the precise triggers of B cell activation in the CNS and the cytokine profiles of CNS invading B cells have yet to be determined. It seems likely that the local production of cytokines and chemokines, such as CXCL13, drives B cell recruitment to the CNS [83], and these pro-inflammatory skewed B cells then propagate inflammation by acting on CNS effector cells, such as microglia and astrocytes, which have been shown to be highly activated in sites of demyelination [84,85,86]. Microglia, in particular, have been found in high numbers at the leading edge of actively demyelinating lesions in MS, suggesting a strong engagement in disease progression. While the clinical data demonstrating a role for B cells in the pathogenesis of MS is very strong, the underlying mechanisms and downstream effects are currently poorly understood. 

### 4.2. Parkinson’s Disease 

Parkinson’s disease (PD) is a neurodegenerative disorder associated with loss of dopaminergic neurons of the substantia nigra pars compacta in the midbrain, resulting in dopamine deficiency in the nigrostriatal pathway [87,88,89]. Symptoms include resting tremor, bradykinesia, and impaired posture and balance. Although the exact etiology of PD is unknown, multiple studies have shown an interplay between the CNS and immune system in PD that may result in a dysregulated immune response in the brain, similar to that seen in MS [90,91,92]. 

For the past few decades, it has been understood that the pathogenesis in PD is associated with inflammatory responses in the brain. Analysis of *post-mortem* brain tissues from patients with PD demonstrated the presence of perivascular deposits of IgG as well as fibrinogen in the striatum, suggesting disruption to the BBB [93,94,95,96,97]. Analysis of patient samples as well as tissue from mouse models of PD have identified CD8+ and CD4+ T cell infiltration into the CNS but little evidence of B cells in the CNS parenchyma [98]. Although there is little evidence of widespread CD19+ or CD20+ B cells presence in the CNS of patients with PD, increased levels of antibodies against CNS proteins have been reported [99,100], suggesting a role for B lineage cells in disease progression. For example, B cell activation can lead to antibody secretion in an inflammatory setting, involving pattern recognition receptor or cytokines released by immune cells. An alternative antibody mediated pathogenic pathway may be the ability of antibodies to initiate or propagate inflammatory reactions by activating the complement system that may result in death of the target cells. Furthermore, activation of the complement system can also lead to microglial activation that can contribute to neuronal loss. Indeed, specific antibodies against dopaminergic neurons have been found in patients with PD [90,98,99], and these antibodies have been found to colocalize with dopaminergic neurons that are closely associated with FcγR+ microglia [92], suggesting microglial activation, antibody-mediated phagocytosis of neurons, and release of pro-inflammatory mediators. The role of antibodies in PD is further supported by pre-clinical animal models of PD showing perivascular inflammation and microgliosis after injection of IgG into the substantia nigra of rats [100]. The same procedure in FcγR(-/-) mice resulted in no microglia activity and nigral injury, suggesting a role for IgG binding in effector cell responses in the CNS. Microglia both respond to as well as propagate inflammation, so it is unclear whether the neuroinflammation was a direct result of IgG presence and whether there is a direct correlation of IgG cytotoxicity and PD disease activity. 

**Figure 3:**
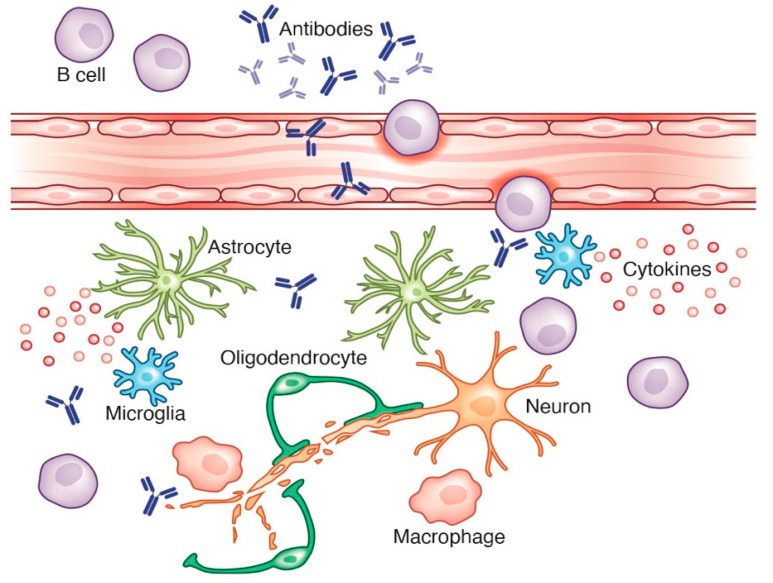
Activated B cells can produce antibodies in an inflammatory setting. Antibodies are capable of activating the complement system, which can activate microglia and astrocytes to release pro-inflammatory factors, leading to neuronal loss. Credit: Katie Vicari.

Parkinson’s disease is associated with changes in the cellular composition of the immune system [91,92,101]. For example, analysis of circulating leukocyte populations, including T, B, and natural killer cells, has shown a small reduction in CD4+ T cells as well as B cells in samples from PD patients [101]. In fact, a continued loss of the T and B cell populations has been reported with clinical stage of disease that was not due to apoptosis [101], suggesting that disease progression may be influenced by alterations to these populations. The means by which PD influences the cell composition of the immune system is unclear but may not be specific to the disease. In other autoimmune diseases, reduced circulating B cell populations were found to be associated with acute inflammation, possibly regulated by pro-inflammatory cytokines [102,103]. Further studies are needed to determine the significance of these findings. Although a PD-specific antigen has not yet been identified, it remains possible that B cells play a role in upregulating or downregulating adaptive immune responses by their secretion of antibodies or cytokines (Figure 3). 

### 4.3. Alzheimer’s Disease 

Alzheimer’s disease (AD) is a progressive neurologic disorder that results in a continuous decline in behavioral, social, and cognitive function as a result of brain atrophy. Alzheimer’s disease pathology is characterized by the presence of ß-amyloid (Aß) plaques [104] and neurofibrillary tangles (NFT) [105]. Despite extensive studies examining the generation, molecular composition, and potential effects of Aß plaques and NFTs in AD, there is no effective treatment for the eventual cognitive decline. In recent years, AD has increasingly been recognized as a neurodegenerative disease characterized by dysregulation of the CNS due to innate and adaptive immune responses. In *post-mortem* AD samples, significant inflammation in the CNS has been observed, mainly by positron emission tomography (PET) targeting microglial activation and reactive astrocyte activity [106,107,108]. Similar to MS pathophysiology, it is increasingly evident that patients with AD exhibit a sustained immune response, possibly due to a disruption of the BBB [109,110]. Deposits of Aß in the CNS vasculature increase cytotoxicity and CNS inflammation that can further disrupt BBB permeability and exacerbate disease [111,112] by allowing cells and molecules from the periphery into the CNS, potentially activating microglia and astrocyte populations to an inflammatory phenotype. This immunological component of AD is further supported by a recent study on AD in mice that showed an improvement in disease symptoms after depletion of B cells or genetic loss of B cells [113]. Additionally, the B cells in AD mice acquired an inflammatory phenotype as shown by the upregulation of pro-inflammatory cytokines, including IL-6, TNFα, and IFNγ. The sustained immune response in AD brains as well as recent animal studies suggesting pathogenic B cells in AD provide compelling evidence of a role for B cells in neuroinflammation and AD pathophysiology. 

## 5. Further Consideration and Conclusions

The efficacy of B cell depletion therapies and extent of antibody involvement in a wide range of neurological diseases provide strong indication that B cells are important targets for therapeutic intervention in CNS disease regardless of whether that role may be in the periphery or in the CNS. The evidence supporting a role for B cells in MS, PD, and AD is strong, while in other neurological diseases such as amyotrophic lateral sclerosis (ALS), it may be a contributing factor in disease progression rather than a disease initiator. ALS is a motor neuron disease with a number of known genetic risk factors including C9orf72 repeat expansion [114,115]. Evidence for an inflammatory involvement includes a high expression of C9orf72 in myeloid cells, including microglia, and an increase in pro-inflammatory factors found in the spinal cords of ALS models, like the mutant Superoxide dismutase 1 gene (SOD1) mice [116]. Furthermore, autoantibodies to CNS antigens have been found in the CSF and serum of patients with ALS [117], suggesting an engagement of the immune system in the progression of the disease. However, a recent study demonstrated that disease development occurred in the mSOD1 model despite a lack of B cells, arguing against the role of B cells in ALS [118]. The SOD1 model, however, has a number of limitations and further studies are clearly warranted to determine the precise role of autoantibodies or B cell-derived cytokines in the pathogenesis of ALS.

Although, in general, B cell function in neurological diseases has not been extensively studied to date, the successes of anti-CD20-depleting antibody treatment in MS are encouraging and demonstrate a clear need to further investigate immunoregulatory functions both within and outside of the CNS in the setting of neurodegenerative diseases. It is possible that B cells do not play a direct role in disease; rather, their secreted factors act on other effector cells that directly affect pathogenesis. A potential model for a B cell driven mechanism is one in which peripheral B cells secrete immunomodulatory molecules, such as cytokines or antibodies, and these molecules invade the CNS by way of a dysfunctional BBB. Alternatively, as shown in the setting of MS, B cells themselves may infiltrate the CNS and directly contribute to glial reactivity or neuronal damage by secretion of inflammatory proteins. These perspectives compel us to further decipher the neuro-immune crosstalk that will surely provide further guidance to not only enhance the efficacy of current B cell-targeted treatments but also discover the next generation of therapeutics. 

## Data Availability

Not applicable.
